# Alterations in the Oral Microbiome Associated With Diabetes, Overweight, and Dietary Components

**DOI:** 10.3389/fnut.2022.914715

**Published:** 2022-07-06

**Authors:** Abeer Shaalan, Sunjae Lee, Catherine Feart, Esther Garcia-Esquinas, David Gomez-Cabrero, Esther Lopez-Garcia, Martine Morzel, Eric Neyraud, Fernando Rodriguez-Artalejo, Ricarda Streich, Gordon Proctor

**Affiliations:** ^1^Faculty of Dentistry, Oral and Craniofacial Sciences, Centre for Host Microbiome Interactions, King’s College London, London, United Kingdom; ^2^School of Life Sciences, Gwangju Institute of Science and Technology, Gwangju, South Korea; ^3^Univ. Bordeaux, Inserm, BPH, UMR 1219, Bordeaux, France; ^4^Department of Preventive Medicine and Public Health, Universidad Autónoma de Madrid and CIBERESP, Madrid, Spain; ^5^Cardiovascular and Nutritional Epidemiology Group, IdiPAZ (La Paz University Hospital-Universidad Autónoma de Madrid), Madrid, Spain; ^6^Translational Bioinformatics Unit, Navarrabiomed, Complejo Hospitalario de Navarra (CHN), Universidad Pública de Navarra (UPNA), IdiSNA, Pamplona, Spain; ^7^Biological and Environmental Sciences and Engineering Division, King Abdullah University of Science and Technology (KAUST), Thuwal, Saudi Arabia; ^8^Institutos Madrileno de Estudios Avanzados (IMDEA)-Food Institute, Madrid, Spain; ^9^STLO, INRAE, Institut Agro, Rennes, France; ^10^Centre des Sciences du Goût et de l’Alimentation, AgroSup Dijon, CNRS, INRAE, Université de Bourgogne Franche-Comté, Dijon, France

**Keywords:** salivatype, oral microbiome, diabetes, Mediterranean diet, obesity, biomarker, saliva

## Abstract

The Mediterranean diet (MedDiet) represents the traditional food consumption patterns of people living in countries bordering the Mediterranean Sea and is associated with a reduced incidence of obesity and type-2 diabetes mellitus (T2DM). The objective of this study was to examine differences in the composition of the oral microbiome in older adults with T2DM and/or high body mass index (BMI) and whether the microbiome was influenced by elements of a MedDiet. Using a nested case-control design individuals affected by T2DM were selected from the Seniors-ENRICA-2 cohort concurrently with non-diabetic controls. BMI was measured, a validated dietary history taken, and adherence to a Mediterranean diet calculated using the MEDAS (Mediterranean Diet Adherence Screener) index. Oral health status was assessed by questionnaire and unstimulated whole mouth saliva was collected, and salivary flow rate calculated. Richness and diversity of the salivary microbiome were reduced in participants with T2DM compared to those without diabetes. The bacterial community structure in saliva showed distinct “signatures” or “salivatypes,” characterized by predominance of particular bacterial genera. Salivatype 1 was more represented in subjects with T2DM, whilst those with obesity (BMI ≥ 30 kg/m^2^) had a predominance of salivatype 2, and control participants without T2DM or obesity had an increased presence of salivatype 3. There was an association of salivatype 1 with increased consumption of sugary snacks combined with reduced consumption of fish/shellfish and nuts. It can be concluded that the microbial community structure of saliva is altered in T2DM and obesity and is associated with altered consumption of particular food items. In order to further substantiate these observations a prospective study should be undertaken to assess the impact of diets aimed at modifying diabetic status and reducing weight.

## Introduction

Human salivary glands secrete approximately 1 L of saliva each day, carrying large numbers of bacteria (approximately 10^8^ per ml) from the mouth into the gastrointestinal tract, resulting in some colonization of the lower gut in both diseased and healthy subjects (1, 2). The microbial composition of saliva reflects the colonization of many oral surfaces and has been found to be inherently stable and resilient, features which resist dysbiosis (3). However, changes in the composition of the oral microbiome have been observed in oral disease, including dental caries and periodontal disease, and in systemic disease, including obesity, and type-2 diabetes mellitus (T2DM) (4, 5).

The core oral microbiota does not appear to greatly depend on diet as an energy source since consortia of oral bacteria are adapted to the digestion of host glycoproteins rather than exogenous food, which remains in the mouth for relatively short periods of time before being swallowed (6). However, persistent, increased sugar consumption does cause dysbiosis, with a shift to a saccharolytic, acidogenic, and aciduric microbiota (3). Although no difference in the oral microbiome was found between subjects on a vegan, lacto-vegetarian or omnivorous diet (7), people consuming a Mediterranean diet (MedDiet), which represents the traditional food consumption patterns in regions bordering the Mediterranean Sea, were found to have reduced levels of oral disease (periodontopathic) associated bacteria in the mouth (8).

The MedDiet pattern of eating, places an emphasis on higher consumption of unprocessed nuts, fruits, vegetables and legumes and lower consumption of animal source foods containing higher levels of saturated fats or highly processed foods with added sugars (9). Greater adherence to a MedDiet has been associated with reduced incidence of cardiovascular disease, obesity and T2DM (9, 10). In a recent study of over 21,000 UKBiobank participants we found that increased adherence to a MedDiet, assessed using an adapted (MEDAS) scoring system derived from dietary recalls, was associated with a reduced risk of T2DM (11). Using a mediation analysis, the reduced risk of T2DM associated with the MedDiet was largely accounted for by a reduction in body mass index (BMI), but there was also a smaller, BMI independent, diet contribution to reduced incidence of T2DM (11). The latter may be due to increased consumption of fibers, micronutrients, and bioactive compounds with anti-inflammatory properties, all of which are present in relatively high amounts in many of the foods comprising the MedDiet (12). It may be that this dietary effect is mediated in part by changes in the gut microbiome, since there is evidence from human and mouse studies, that altered composition and function of the gut microbiota can contribute to the development of obesity and T2DM, through altered microbial production of short-chain fatty acids and microbial metabolism of host bile acids, both of which can impact on host glucose metabolism and insulin signaling (13). Obesity and T2DM are associated with gut barrier disruption and increased permeability to bacterial-derived inflammogens such as LPS, leading to increased systemic inflammation (13–15). MedDiet intervention in overweight and obese subjects lowers plasma cholesterol and alters the gut microbiome and metabolome independently of energy intake, with an increased diversity of the gut microbiome being correlated with reduced systemic inflammation as indicated by serum hs-CRP concentrations (16).

Since saliva is relatively easy to collect repeatedly, salivary biomarkers have great potential in monitoring disease activity and adherence to dietary plans, including the MedDiet (17). In the present nested case-control study saliva samples were collected from a group of participants with T2DM and a second group of non-T2DM participants. The aims of the study were to compare the composition of the salivary microbiome in T2DM and non-T2DM groups and determine whether BMI status or MedDiet adherence influenced salivary microbiome composition. We found that the microbial community structure of saliva is altered in T2DM and obesity and is associated with altered consumption of particular food items.

## Materials and Methods

### Population

In this study 121 individuals (67–84 years) were recruited from a population-based cohort on aging, the Seniors-ENRICA-2 cohort in Spain. The cohort was established in order to examine the associations between lifestyle and healthy aging in older adults (18). This study was reviewed and approved by the Ethics Research Committee of the «La Paz» University Hospital (Madrid). The participants provided their written informed consent to participate in this study.”

### Studied Samples

The 121 Individuals included in the study were selected using a nested case-control design. Cases (*n* = 61) were individuals affected by T2DM based on self-reported physician’s diagnosis and/or being on anti-diabetic treatment (oral medication or insulin) at the time of data collection. Controls (*n* = 60) were selected concurrently and were free of diabetes at the time of data collection. For the present study, we used the following information from each participant: sex, age (years)—categorized as old (i.e., 67–75 year) and oldest old (i.e., 76–84 y), BMI (calculated as measured weight in kg divided by the square of measured height in m), T2DM status, smoker status (never, former, current), food consumption, and adherence to the MedDiet.

### Dietary Surveys and Assessment of Adherence to the Mediterranean Diet

Food consumption data were collected using a validated electronic diet history (19). Adherence to the Mediterranean dietary pattern was assessed by calculating the MEDAS score (20). The MEDAS score ranges from 0 to 13, with higher values indicating higher adherence to the Mediterranean diet. We restricted the analysis to the 11 MEDAS items describing the consumption of food groups, namely olive oil, cooking with olive oil, vegetables, fruits, red meat, butter/margarine/cream, carbonated sweet drinks, wine, legumes, fish/shellfish, sweet snacks (confectionary, biscuits, and commercial pastries), and nuts. For each food group, the participants were classified as high or low consumers based on the cut-off points defined for the MEDAS score calculation. High consumers or each “healthy” food item scored 1 on MEDAS and low consumers scored zero, whilst high consumers of the food items red meat, butter/margarine/cream, carbonated sweet drinks and sweet snacks scored zero and low consumers scored 1.

### Oral Health Questionnaire

Oral health was assessed by questionnaire (Supplementary Table 1) containing the following features: mouth condition was scored from very good (1) to poor (5); wearing of dentures (yes or no); number of visits to the dentist, from less than 1, 1, 2 or greater than 2 visits per year (scored 1–4); oral health was scored from 0 to 5 based on presence of the following items, each scoring 1: bleeding gums, painful gums, painful teeth, mouth ulcers, loss of 2 or more teeth in last 2 years.

### Saliva Sampling and Handling

Participants collected unstimulated saliva at home early in the morning after overnight fasting. Drinking water was permitted up to 5 min before saliva collection and participants sat comfortably, tilted their heads slightly downwards, allowed saliva to pool on the floor of the mouths and spat into 40 ml polypropylene tubes. Sampling was performed for 10 min but in case a participant wished to stop before the end of the 10 min, the time was recorded in order to be able to calculate the saliva flow (expressed in mL/min and categorized as follows normal (> or = 0.25 mL/min), low (0.1–0.24 mL/min), or hypofunctional (<0.1 mL/min) (21). Saliva samples were immediately placed on ice, transported to the laboratory, and placed at -80°C as soon as possible (never longer than 4 h on ice). At the end of the collection from the participants, samples were shipped to the analytical facilities on dry ice.

### DNA Extraction and 16S rRNA Gene Pyrosequencing

DNA was extracted from saliva samples using the GenElute Bacterial Genomic DNA extraction kit (NA2120, Sigma-Aldrich) following the manufacturer’s instructions with an additional lysozyme (45 mg/ml) (89833, Thermo Fisher Scientific) incubation step for 30 min at 37°C. DNA purity and integrity were evaluated *via* NanoDrop 7000 Spectrophotometer (Thermo Fisher Scientific, Waltham, MA, United States) and 1% agarose (BIO-41025, Bioline) gel electrophoresis, respectively, and DNA was stored at -80°C for subsequent experiments. The V1-V2 regions of the bacterial 16S rRNA genes were amplified using a 27F-YM and 338R-R forward and reverse tagged primer pairs (Eurofins Genomics) in a polymerase chain reaction (PCR). A negative control (no template) was included on each PCR plate and the ZymoBIOMICS Microbial Community DNA Standard (D6305, Cambridge Bioscience) was added as a sample in the library and used as a positive control to evaluate the quality of the sequencing. The PCR program was: initial denaturation at 98°C for 2 min, followed by 25 cycles of denaturation at 98°C for 10 s, annealing at 55°C for 30 s, and extension at 72°C for 30 s, followed by a final extension at 72°C for 5 min. PCR products were run on a 1% agarose gel with GelRed^§^ Nucleic Acid Gel Stain (41002, Biotium) to confirm successful amplification of the ∼350-bp fragment. The purified amplicon PCR products were quantitated using Quant-iT PicoGreen dsDNA Assay Kit (P7589, Thermo Fisher Scientific) and pooled at equal quantities. In order to achieve successful cluster amplification, the pooled PCR products were further cleaned up using QIAquick PCR Purification columns (28106, Qiagen). Pools were quantified using Qubit dsDNA HS Assay Kit (10606433, Fisher Scientific Ltd.) according to the manufacturer’s instructions. Then, pools were diluted to the same concentration and mixed at the same ratio to get the final sequencing pool. The library was sent to the Genome Centre at Queen Mary University London for multiplex sequencing (Illumina MiSeq) of the tagged samples.

### Bioinformatics and Statistical Analysis

A total of 3,788,644 raw sequences were obtained from MiSeq Illumina sequencing. The Divisive Amplicon Denoising Algorithm (DADA2) pipeline of R software (version 1.8.0) was used for quality-filtering, trimming, error correction, exact sequence inference, chimera removal, and generation of amplicon sequence variant tables (ASV) (22). The final dataset contained an average of 24,621 sequences per sample, leading to a total 6,840 ASVs. Bacterial taxonomic classification was determined by reference to the Human Oral Microbiome Database (HOMD). Diversity indices such as Observed, Shannon, and Inverse Simpson were calculated using the DADA2 R package and compared by Wilcoxon rank-sum tests. For the ordination plot, multi-dimensional scaling (MDS) was performed using Bray-Curtis distances. Group differences by diabetic status on the ordination plot were calculated based on the PERMANOVA test (R vegan package). Contrasted taxa were investigated by R DESeq2 negative binomial tests. The Dirichlet multinomial mixtures method was applied to identify optimal numbers of distinct clusters representing unique oral microbiome communities (23).

Participant cohort characteristics, dietary intake, and health characteristics were expressed as means ± SD and compared by Chi-square tests.

## Results

### Participant Characteristics

Table 1 summarizes the characteristics of the participants included in the current study. There were no differences in gender or age distributions; smoking and MEDAS score between the T2DM and non-T2DM groups (*P* > 0.05). In total, 21 out of 56 diabetic participants had obesity and 8 of 56 were lean (BMI < 25 kg/m^2^) whilst 14 out of 60 non-T2DM participants had obesity and 16 out of 60 were lean (Table 1). Oral health was similar in T2DM and non-T2DM participants as revealed by the oral health questionnaire (Supplementary Table 1). Salivary flow rates varied from 0.005 to 1 ml/min and mean salivary flow rate was not reduced in the T2DM group compared to controls, but 20 out of 61 in the T2DM group had salivary gland hypofunction (<0.1 ml/min) compared 12 out of 60 non-T2DM participants (Table 1 and Supplementary Table 1).

**TABLE 1:** Summary of the characteristics of the participant cohort and comparison of participants with and without T2DM.

Characteristics	T2DM (*n* = 61)		Non-T2DM (*n* = 60)		*P*-value
	*N*	%	*N*	%	
**Age**					
Mean		74.3 ± 4.4		74.3 ± 4.9	
Old (67–75 yr)	36	59.0	34	56.7	0.938
Oldest old (76–84 yr)	25	41.0	26	43.3	
**Gender**					
Male	35	57.4	25	41.7	0.122
Female	26	42.6	35	58.3	
**Smoking**					
Never	26	42.6	32	53.3	0.438
Former	33	54.1	26	43.3	
Current	2	3.3	3	5.0	
**MEDAS score**					
High (≥7)	37	60.7	38	63.3	0.908
Low (≤6)	24	39.3	22	36.7	
**BMI**					
Normal (<25)	8	13.1	16	26.7	0.13
High (25–29)	27	44.3	30	50.0	
Obese (≥30)	21	34.4	14	23.3	
Missing	5	8.2			
**Saliva flow (ml/min)**					
Normal (≥0.25)	19	30.6	23	39	0.163
Low (0.1–0.24)	22	35.5	25	42.3	
hypofunction (<0.1)	21	33.9	11	18.6	

*Statistical assessment used Chi-square test.*

### Intake of Dietary Components Comprising the MedDiet Assessed Scores

The numbers of high and low consumers of 11 food groups separated by T2DM status are presented in Table 2. The dietary patterns were overall comparable between diabetic and non-diabetic individuals except for one food group, “sweets snacks” (confectionary and commercial pastries) which showed a higher proportion of high consumers among non-T2DM subjects. Correlation analysis of selected food items from the MEDAS score indicated that some were better correlated with T2DM or high BMI (Supplementary Figure 1).

**TABLE 2:** Comparison of MEDAS scores of adherence to a Mediterranean diet in groups of participants with and without T2DM.

	T2DM (*n* = 61)		Non-T2DM (*n* = 60)		*P*- value
	N	%	N	%	
**Olive oil**					
H = 4 tbsp/day	8	13.1	9	15	0.971
L <4 tbsp/day	53	86.9	51	85	
**Olive oil cooking**					
Yes	59	96.7	57	95	0.985
No	2	3.3	3	5	
**Vegetables**					
H = 2 servings/day	1	1.6	4	6.7	0.351
L < 2 servings/day	60	98.4	56	93.3	
**Fruit**					
H = 3 units/day	29	47.5	19	31.7	0.110
L < 3 units/day	32	52.5	41	68.3	
**Red meat**					
H = 1 serving/day	10	16.4	7	11.7	0.627
L < 1 serving/day	51	83.6	53	88.3	
**Butter, margarine, cream**					
H = 1 serving/day	5	8.2	7	11.7	0.738
L < 1 serving/day	56	91.8	53	88.3	
**Carbonated drinks (soda)**					
H = 1/day	10	16.4	6	10	0.442
L < 1/day	51	83.6	54	90	
**Wine**					
H = 7 glasses/week	9	14.8	10	16.7	0.969
L < 7 glasses/week	52	85.2	50	83.3	
**Legumes**					
H = 3 servings/week	8	13.1	11	18.3	0.590
L < 3 servings/week	53	86.9	49	81.7	
**Fish/Shellfish**					
H = 3 servings/week	17	27.9	17	28.3	1.000
L < 3 servings/week	44	72.1	43	71.7	
**Sweet snacks**					
H = 3 times/week	23	37.7	35	58.3	0.0367*
L < 3 times/week	38	62.3	25	41.7	
**Nuts**					
H = 3 servings/week	13	21.3	15	25	0.791
< 3 servings/week	48	78.7	45	75	

Statistical assessment used Chi-square test (*p < 0.05).

### Salivary Microbiome Richness and Diversity in Type-2 Diabetes Mellitus and Non-type-2 Diabetes Mellitus Subjects

Following assessment of the quality of isolated DNA, we generated a total of 109 samples of oral metagenomics data based on 16S rDNA amplicon sequencing. Rarefaction curves were plotted to verify the efficiency of the sequencing process (Supplementary Figure 2 and Supplementary Table 2). Using the R Dada2 software package, we pre-processed short reads and checked observed amplicon sequence variants (ASVs) by the sequencing depths, observing no increase in the number above our minimal sequencing depth; those samples (*n* = 5) with less than the minimal depth were excluded from the analysis (Supplementary Figure 2). Therefore, the final number of samples for assessment of the microbial community was *n* = 99.

**FIGURE 1:**
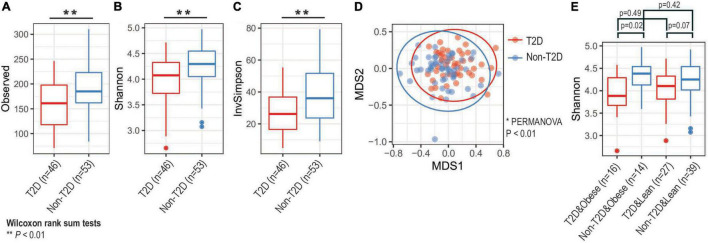
Analysis of microbial diversity. Based on **(A)** observed, **(B)** Shannon, and **(C)** inverse Simpson diversity, we found that alpha-diversity of oral microbiota decreased among diabetic groups (** Wilcoxon rank sum tests *p*-value < 0.01). **(D)** Principal coordinate analysis (PCoA) identified that oral microbiota compositions were distinct by diabetic status (* PERMANOVA test *p*-value < 0.01). Large circles represent 95% confidence ellipse for each group. **(E)** Obesity status did not significantly change alpha-diversity changes (Wilcoxon rank sum tests). T2D, type 2 diabetes.

The microbial diversity of the oral microbiome was determined in T2DM and non-T2DM participants (Figure 1). Based on the number of observed species, the Observed diversity, Shannon, and Inverse Simpson indices, indicated significantly decreased alpha-diversity of the oral microbiome in T2DM participants (Wilcoxon rank sum tests *p* < 0.05 or *p* < 0.01; Figures 1A–C). Overall, this was also true when the analysis was stratified by weight status (Figure 1E), although the difference reached statistical significance only for participants with obesity. We also performed multi-dimensional scaling of Bray-Curtis distances of all samples and observed distinct separations of diabetic and non-diabetic groups (PERMANOVA *p*-value < 0.01; Figure 1D).

### Bacterial Community Structure of the Salivary Microbiome in Type-2 Diabetes Mellitus and Non-type-2 Diabetes Mellitus Groups

**FIGURE 2:**
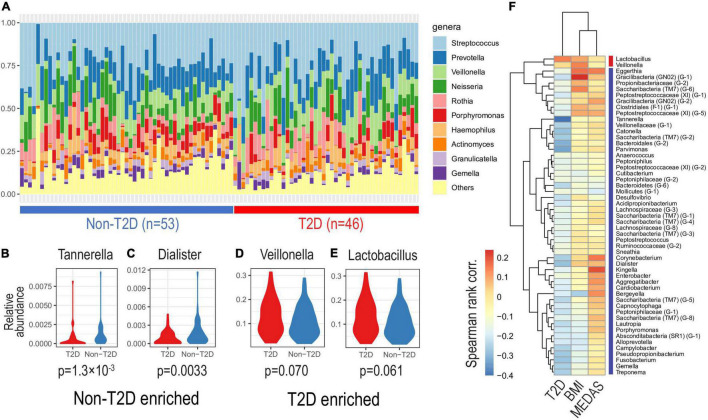
Oral microbiota composition by diabetic status. Based on relative abundance at the genus level, we investigated oral microbiota compositions according to diabetic status. **(A)** The top-10 most abundant genera of different salivatypes. Genera enriched in non-diabetic **(B,C)** and diabetic **(D,E)** subjects (Wilcoxon tests *p*-values are shown). **(F)** Correlations of genus abundances with T2D, BMI, and MedDiet status as shown by the MEDAS score (Spearman’s rank correlation).

The relative contributions of the more dominant bacterial genera were not significantly different between the T2DM and non-T2DM groups although there was a trend suggesting increased abundance of *Veillonella* and *Lactobacillus* in T2DM (Figures 2A,D,E). There was a significant reduction in the proportion of “Others,” the grouped less-dominant genera, as exemplified by *Tannerella* and *Dialister* (Wilcoxon rank sum tests, one-sided, *p*-values < 0.005; see Figures 2B,C and Supplementary Table 3). Based on correlation analysis, we found that many taxa negatively correlated with T2DM status were positively correlated with MedDiet status, possibly reversing T2DM-specific microbial community (Figure 2F).

**FIGURE 3:**
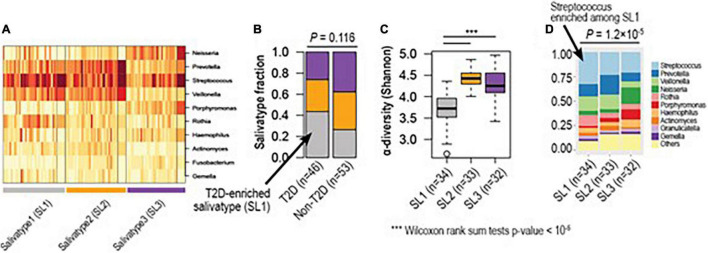
Hidden microbial community structure (salivatype) among diabetic and non-diabetic groups. **(A)** Based on unsupervised clustering, we identified three clusters of different microbial compositions, named salivatype 1, 2, and 3. Salivatype 1 was enriched in *Streptococcus*, salivatype 2 enriched in *Prevotella*, and salivatype-3 enriched in *Neisseria*. **(B)** Salivatype-1 was enriched among diabetic subjects compared to non-diabetic subjects (Chi-square tests *p*-value = 0.116). **(C)** Salivatype-1 was the lowest in alpha diversity (*** Wilcoxon rank sum tests *p*-values < 1e-5). **(D)** The top-10 most abundant genera of different salivatypes. *Streptococcus* was highly enriched in salivatype1, as compared to other salivatypes (Wilcoxon rank sum tests *p*-value = 1.2 × 10^–5^). T2D, Type 2 diabetes mellitus.

To identify optimal numbers of distinct clusters representing unique oral microbiome communities, we applied an unsupervised clustering method, Dirichlet multinomial mixtures, which has been applied to identify “enterotype,” which represents unique gut microbial community structure associated with diets, lifestyle and dysbiosis (24) (Figure 3A). Here we observed three distinct clusters herein called salivatypes. Salivatype-1 was enriched with the genera *Streptococcus*, *Rothia* and *Veillonella* whereas salivatype-2 was enriched in *Prevotella*, *Actinomyces* and *Veillonella* and salivatype-3 with *Neisseria* and *Porphyromonas* (Figures 3A,D). Interestingly, we found significant changes of salivatype proportions between the T2DM and non-T2DM groups with salivatype-1 increased among diabetic participants, and salivatype 3 was increased among non-diabetic participants (Figure 3B and Supplementary Table 4). Salivatype 2 was enriched in obese subjects. Salivatype 1 had a reduced diversity compared to salivatypes 2 and 3 (Figure 3C), and there was a reduced proportion of the less dominant genera, grouped as “Others” (Figure 3D). Salivatype 1 when present in non-T2DM, obese, and non-obese subjects had a similar decreased diversity (Supplementary Figure 3).

### Relative Abundance of Microbiome Genera in Participants of Differing Body Mass Index Status or Adherence to a Mediterranean Diet

**FIGURE 4:**
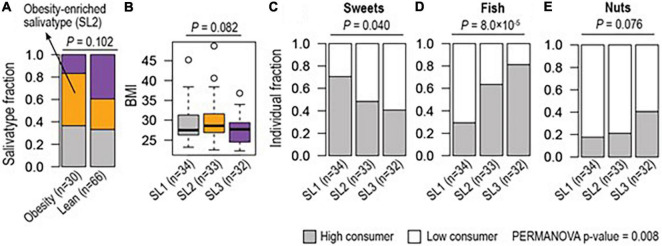
Different enrichment of salivatype (SL) in obesity and in relation to consumption of food groups. **(A)** Salivatype-2 was enriched among obese (≥ 30 BMI) subjects compared to lean (< 25 BMI) subjects (Chi-square test *p*-value = 0.102). **(B)** Salivatype-2 was associated with a high median BMI (ANOVA *p*-value = 0.082). Together, **(C)** sweet snacks, **(D)** fish, and **(E)** nuts were differently consumed among subjects with different salivatypes (Chi-square tests, *p*-values < 0.1) and their coordinate changes were associated with salivatypes (PERMANOVA *p*-value = 0.008).

Figures 4A,B show the relative abundance of the microbiome salivatypes in obese (*n* = 30) compared to non-obese (*n* = 66) participants and the median BMI values of participants showing each salivatype. There is a trend of reduction in salivatype 3 in obese subjects, accompanied by an increased abundance of salivatype 2 compared with lean participants.

Salivatype 2 tended to be associated with a higher (7–11) MEDAS score whilst a low (≤ 6) MEDAS score tended to be associated with a salivatype 1 microbiome, but these were not statistically significant differences. When single dietary items included in the MEDAS score were considered, there was a lower consumption of sweet snacks in participants with increased presence of salivatype 1. The items on eating of fish and nuts were greater in those with increased presence of salivatype 3. Collectively these food (i.e., sweets, fish, and nuts) items were statistically significantly different between the salivatypes (Figures 4C–E).

## Discussion

In this study of older adults, we observed a reduced richness and diversity of the salivary microbiome in T2DM, a result which agrees with some previous studies (4, 5, 25). However, T2DM has been associated with an increased prevalence of periodontal disease and subjects with the latter can show an increased oral microbial diversity and richness (25). We used a questionnaire to assess the oral health of participants and it asked about a diagnosis of periodontal disease, the presence of bleeding and painful gums, and loss of 2 or more teeth in the last 2 years; the responses to these questions suggested that levels of periodontitis were similar in both the T2DM and non-T2DM groups.

The most abundant genera in this study, *Streptococcus, Prevotella, Veillonella, Neisseria*, *Rothia*, *Porphyromonas*, and *Haemophilus* have similarly been observed previously (7, 26, 27). Comparison of the relative abundances of bacterial genera in T2DM and non-T2DM participants suggested that the major genera *Veillonella* and *Lactobacillus* tended to be increased but this did not reach statistical significance. Such changes might implicate the over-production of lactate in the microbial community of subjects with T2DM, due to altered host metabolism. There were also decreases in the relative presence of non-dominant genera in T2DM, which needs further investigation of their roles as keystone species.

Unsupervised clustering of the microbiome sequencing data suggests 3 salivatypes characterized by distinct relative abundances of some major bacterial genera. Recently, similar unsupervised clustering was applied to the gut (fecal) microbiome and microbial community signatures were identified, suggesting distinct communities of the gut microbiome, referred to as enterotypes (28). Co-occurring and co-excluding genera of the salivatypes identified in the present study, show many similarities with the distinct clustering of the salivary microbiome described previously in healthy subjects (7). We found that salivatype 1 (increased *Streptococcus*, *Veillonella*, *Rothia*) was predominant in subjects with T2DM, which supports a similar finding of combined increases in *Streptococcus*, *Veillonella*, and *Rothia* in a previous study of diabetic compared to normoglycemic subjects (5). The increases in genera containing aciduric species may be due to altered host metabolism of sugar to lactate, and further metabolism of lactate by species in the genus *Veillonella* may contribute to the resilience of the oral microbiome to acidification (3, 29). There was predominance of a different microbiome-related salivatype (SL2) in obese subjects, compared to lean subjects. Salivatype 2 is characterized by increased *Prevotella* and *Actinomyces*. It has previously be found that obesity alters the composition and diversity of the oral microbiome in patients with T2DM independently of glycemic control (4), and that *Prevotella* and *Actinomyces* species are increased in the salivary microbiome of obese adults without periodontal disease (30).

Although the (MEDAS) scoring of adherence to a Mediterranean diet did not differ between T2DM and non-T2DM groups or subjects with and without obesity, a combination of three dietary items (low sugar snacks, high fish/shellfish and high nuts) were associated with an altered microbial community structure. Subjects consuming fewer sweet snacks, showed an enrichment of salivatype 1, whilst those consuming more fish and nuts, showed enrichment in salivatype 3. An association of salivatype 1 with low sugary snacks and T2DM seems logical since sugary snacks were consumed less frequently in the T2DM group.

The present study has some limitations, including the selection of subjects based on diabetic status and the absence of a clinical assessment of oral health. In a future study saliva samples should be collected longitudinally from subjects with T2DM, subjects with obesity and matched control subjects, before and after adoption of a Mediterranean diet. We used 16SrRNA gene amplification and sequencing, which has been shown to capture the majority of bacterial taxa in many previous studies of the oral microbiome (31). We did not use whole genome sequencing in this study; although there are a number of advantages over 16SrRNA gene amplification sequencing, including bacterial classification down to species and strain level, characterization of fungal species and the microbial resistome (32); a further consideration was that saliva sample volume was limited, since other omics analyses were undertaken (33). A future longitudinal study should utilize genome shotgun metagenomic sequencing of saliva, rather than 16SrRNA gene amplification and sequencing, which would provide more detail of the structure and function of the microbial community, especially the community of minor microbial taxa, in T2DM, obesity and its response to components of the MedDiet. If salivary microbiome biomarkers are validated in such a study then these could be usefully employed in monitoring adherence to a MedDiet and the course of metabolic disease.

## Data Availability Statement

The datasets presented in this study can be found in the online repository https://www.ebi.ac.uk/ena, PRJEB51205.

## Ethics Statement

The studies involving human participants were reviewed and approved by the Ethics Research Committee of the «La Paz» University Hospital (Madrid). The participants provided their written informed consent to participate in this study.

## Author Contributions

EN, CF, FR-A, EL-G, GP, and MM conceived and designed the study. AS and RS performed saliva analysis. SL, AS, and DG-C performed statistical analyses. EG-E, FR-A, and EL-G provided epidemiological data and sampled saliva. AS, GP, and SL drafted the manuscript. All authors revised the manuscript and approved its final version.

## Conflict of Interest

The authors declare that the research was conducted in the absence of any commercial or financial relationships that could be construed as a potential conflict of interest.

## Publisher’s Note

All claims expressed in this article are solely those of the authors and do not necessarily represent those of their affiliated organizations, or those of the publisher, the editors and the reviewers. Any product that may be evaluated in this article, or claim that may be made by its manufacturer, is not guaranteed or endorsed by the publisher.

## Supplementary Material

FIGURE S1 Odds ratios of intake food items by different MEDAS, T2D, and BMI status. (Fisher’s tests *p*-values shown) T2D: Type 2 diabetes mellitus. Note that a higher MEDAS score indicates a low consumption of sweet snacks (sweet), red meat, butter and soda and a high consumption of all other food items.

FIGURE S2 Rarefaction curves of amplicon sequence variants (ASVs), showing depth of 16SrRNA gene sequencing of saliva samples.

FIGURE S3 Salivatype-1 when present in non-T2DM, obese and non-obese subjects had a similar decreased alpha-diversity.
