# Supplementary material for: Alterations in the Oral Microbiome Associated With Diabetes, Overweight, and Dietary Components
Source: Front Nutr. 2022 Jul 6;9:914715. doi: 10.3389/fnut.2022.914715 (PMC9298547; doi:10.3389/fnut.2022.914715)
Supplement: Supplementary file 1 [file Data_Sheet_1.docx]

Supplementary figure captions and legends

Figure S1. Correlation of food items with MEDAS, T2DM and BMI.

Odds ratios of food items by MEDAS score (<7 vs ≥7), diabetes status (presence vs absence), and BMI status (lean vs obesity). Fisher’s tests p-values shown. T2D: Type 2 diabetes mellitus. Note that a low consumption of sweet snacks (sweet), red meat, butter and soda and a high consumption of all other food items each score 1 in the MEDAS.

Figure S2. Rarefaction curves of amplicon sequence variants (ASVs), showing depth of16SrRNAgene sequencing of saliva samples.

Figure S3. Salivatype-1 when present in T2DM, non-T2DM, obese and non-obese subjects had a similar decreased alpha-diversity.
