# Supplementary material for: Alterations in the Oral Microbiome Associated With Diabetes, Overweight, and Dietary Components
Source: Front Nutr. 2022 Jul 6;9:914715. doi: 10.3389/fnut.2022.914715 (PMC9298547; doi:10.3389/fnut.2022.914715)
Supplement: Supplementary file 2 [file Table_1.pdf]

Table S1. Dataset for participant characteristics (oral health).

All counts or mean values are shown with percentages or standard deviations, respectively. [1]Mouth condition was scored from 1 (v.good) to 5 (poor). [2]Dental visits per year. [3]Oral health was scored based on the presence of the following symptoms: bleeding gums, painful gums, painful teeth, mouth ulcers, and loss of 2 or more teeth during the last two years.

	T2DM (N = 61)	Non-T2DM (N = 60)
**Mouth condition[1] (score)**	2.54 (±0.808)	2.45 (±0.622)
**Denture wearing**		
no	3 (4.9%)	4 (6.7%)
yes	58 (95.1%)	56 (93.3%)
**Dentist visits[2]**	3.82 (±0.533)	3.78 (±0.555)
**Periodontal disease (score)[3]**	3.41 (±0.761)	3.23 (±0.789)
**Difficulties of eating**		
no	14 (23.0%)	17 (28.3%)
yes	47 (77.0%)	43 (71.7%)
**Flow rates**	0.186 (0.154)	0.230 (0.179)
**Oral health (score)**	1.30 (±1.05)	0.983 (±0.854)
