# Supplementary material for: Alterations in the Oral Microbiome Associated With Diabetes, Overweight, and Dietary Components
Source: Front Nutr. 2022 Jul 6;9:914715. doi: 10.3389/fnut.2022.914715 (PMC9298547; doi:10.3389/fnut.2022.914715)
Supplement: Supplementary file 3 [file Table_2.pdf]

Table S2. Statistics of sequencing reads. Number of sequencing reads were checked after filtering, denoising, merging, and removal of chimera.

Participant	input sequence	filtered sequence	denoised forward	denoised reverse	merged	removing chimera	kept percentage	Note
AS-131	26066	23909	23074	23403	20812	18401	70.59387708	
AS-132	23969	22019	21487	21635	19391	18079	75.42659268	
AS-133	25525	23369	22726	23047	20878	19196	75.20470127	
AS-134	22327	20477	20004	20260	18704	16439	73.62834237	
AS-135	22243	20446	19709	19965	17812	16082	72.30139819	
AS-136	25247	23327	22547	22868	20629	19156	75.87436131	
AS-137	22483	20348	19781	20071	17928	15818	70.35537962	
AS-138	28777	26408	26017	26161	23871	20099	69.84397262	
AS-141	30618	28505	27557	28025	24501	19908	65.02057613	
AS-142	23841	22060	21680	21870	20207	15681	65.77324777	
AS-143	29226	27300	26799	27049	24932	22231	76.06583179	
AS-144	34405	31760	30887	31443	27515	21564	62.67693649	
AS-145	34590	31918	31177	31551	27942	20727	59.92194276	
AS-146	30055	27812	26824	27315	23535	20187	67.16686076	
AS-147	25118	23151	22799	23020	21404	18346	73.03925472	
AS-150	25221	23096	22062	22552	18874	15158	60.10070973	
AS-151	26735	24444	23440	23976	20296	16004	59.86160464	
AS-154	29749	27262	26781	26954	24753	22114	74.33527177	
AS-155	31068	28537	27680	28027	24471	20350	65.50148062	
AS-156	32489	29651	28851	29258	25735	22195	68.31542984	
AS-157	26400	24302	23440	23969	21034	19128	72.45454545	
AS-158	25833	23544	22984	23224	21304	19169	74.20353811	
AS-160	22505	20520	20102	20214	17991	14147	62.86158631	
AS-161	27114	24898	24090	24474	21415	17524	64.63081803	
AS-162	33902	31077	30183	30603	27361	23839	70.3173854	
AS-163	32183	29431	28621	28962	25601	21653	67.28086257	
AS-164	33134	30705	30143	30354	28125	24208	73.06090421	
AS-165	28875	26739	25952	26359	23727	20830	72.13852814	
AS-166	31665	28982	28040	28439	25189	21319	67.32670141	
AS-168	41821	38630	38303	38464	37363	34614	82.76703092	
AS-169	79782	73324	71906	72375	65402	55120	69.08826552	
AS-170	32587	30407	29769	30071	27931	23803	73.04446558	
AS-171	35447	32702	32233	32476	30677	26702	75.32936497	
AS-172	41402	38491	37965	38322	36242	30882	74.59059949	
AS-173	41108	38022	36982	37437	33384	29208	71.05186338	
AS-174	34418	31617	30873	30991	27880	23941	69.5595328	
AS-175	36834	34164	32855	33498	27511	21398	58.09306619	
AS-176	38419	35888	35001	35300	31525	28706	74.71823837	
AS-177	44088	40946	39464	40300	34975	28953	65.67093087	
AS-178	37859	34625	33189	33825	29070	24054	63.53575108	
AS-179	36673	33117	32234	32711	29550	23562	64.24890246	
AS-180	43374	39778	38588	39098	34593	29314	67.58426707	

AS-181	47147	43338	42483	42911	38794	32485	68.90152078	
AS-182	36132	32911	32052	32481	28886	23968	66.33455109	
AS-183	39113	35726	34949	35395	32163	25052	64.05031575	
AS-184	37477	34692	33776	34255	30426	25619	68.35926035	
AS-185	39295	36112	34712	35510	30802	25081	63.82745896	
AS-186	31911	29242	28288	28709	25189	22061	69.13290088	
AS-189	34459	31334	30269	30728	26703	22960	66.62990801	
AS-190	51621	46952	45205	46069	39230	32450	62.86201352	
AS-191	43198	39549	38182	38815	34065	28178	65.22987175	
AS-192	30593	27881	27091	27434	24448	18999	62.10244174	
AS-193	60080	54894	53449	54350	48537	40199	66.90912117	
AS-194	38686	35222	34329	34825	31111	26863	69.43855658	
AS-195	61557	56199	55064	55631	51358	44649	72.5327745	
AS-196	45210	40982	40301	40705	37970	31675	70.0619332	
AS-197	43983	40323	39284	39779	35618	31252	71.05472569	
AS-198	52009	47582	46452	47009	41855	37143	71.41648561	
AS-199	47495	41194	40507	40870	37737	30918	65.09737867	
AS-200	47214	42486	41614	42095	38744	32254	68.31448299	
AS-201	38863	35953	35123	35481	32210	28669	73.76939505	
AS-202	33572	29731	29141	29457	27551	23878	71.12474681	
AS-203	42537	38810	38043	38331	34437	28370	66.6948774	
AS-206	36770	33286	32429	32806	28789	25297	68.7979331	
AS-207	31791	28995	28142	28649	25389	21985	69.15479224	
AS-208	25327	23257	23080	23185	22789	22679	89.54475461	
AS-209	38536	34438	33430	34035	29445	26732	69.36890181	
AS-210	38309	34900	33808	34294	30263	27835	72.65916625	
AS-211	41923	38913	37989	38432	35235	33494	79.89409155	
AS-212	30967	28650	27967	28303	25746	23012	74.31136371	
AS-213	37980	34960	34544	34657	33059	32159	84.67351237	
AS-214	40631	37516	36393	36940	32908	29020	71.42329748	
AS-215	46233	42957	41868	42550	38343	32830	71.00988471	
AS-217	40075	37146	36146	36591	32947	30172	75.28883344	
AS-218	41751	38550	38083	38309	36488	32837	78.64961318	
AS-219	44068	40681	40068	40447	37890	31611	71.73232277	
AS-220	6206	5735	5714	5719	5701	5091	82.03351595	Removed
AS-221	4950	4581	4355	4449	3910	3771	76.18181818	Removed
AS-222	7329	6697	6422	6500	5710	5539	75.57647701	Removed
AS-223	6176	5531	5267	5351	4543	4506	72.95984456	Removed
AS-224	6375	5845	5655	5687	5195	4969	77.94509804	Removed
AS-225	47740	43664	42956	43334	39212	33538	70.25136154	
AS-226	57616	52339	50910	51590	46243	40572	70.41793946	
AS-228	81921	75205	73255	74442	65594	54033	65.95744681	
AS-229	52694	46947	45812	46380	41902	39600	75.15087107	
AS-230	53343	47869	46540	47190	42297	37276	69.87983428	
AS-231	46456	42526	41346	41885	37236	31104	66.9536766	
AS-232	40886	37489	36805	37095	34171	32292	78.98058015	
AS-233	34745	31580	30138	30794	25969	23188	66.73766009	
AS-234	36688	33591	32694	33106	30274	27260	74.30222416	

AS-235	40550	37142	36363	36701	33540	30478	75.16152898	
AS-236	53270	48944	47896	48381	44775	39699	74.5241224	
AS-237	39138	35737	35025	35273	32660	29732	75.96709081	
AS-238	31360	28586	27693	28048	24418	21383	68.18558673	
AS-239	31640	28842	28435	28571	26701	24562	77.62958281	
AS-240	38323	35165	33980	34446	29564	24748	64.57740782	
AS-241	29985	27842	27281	27473	25302	23383	77.9823245	
AS-242	32114	29531	29286	29369	28237	27567	85.8410662	
AS-243	41240	38303	37480	37905	34344	30981	75.12366634	
AS-244	41682	38405	37556	38095	34681	31833	76.37109544	
AS-245	35072	32425	31473	31917	28674	26015	74.17598084	
AS-246	38999	36029	35397	35660	33000	28993	74.34293187	
AS-247	34138	31761	31291	31517	29620	27396	80.25074697	
AS-248	39418	36706	35863	36191	33153	30884	78.34999239	
AS-249	6062	5561	5368	5407	4902	4794	79.08281095	Removed
AS-250	7059	6486	6198	6330	5569	5497	77.87221986	Removed
AS-251	6649	6109	5896	5945	5292	5223	78.55316589	Removed
AS-252	3610	3290	3123	3195	2732	2708	75.01385042	Removed
AS-253	3522	3184	3105	3130	2911	2911	82.65190233	Removed
