# Supplementary material for: Alterations in the Oral Microbiome Associated With Diabetes, Overweight, and Dietary Components
Source: Front Nutr. 2022 Jul 6;9:914715. doi: 10.3389/fnut.2022.914715 (PMC9298547; doi:10.3389/fnut.2022.914715)
Supplement: Supplementary file 4 [file Table_3.pdf]

Table S3. Increased or decreased genera in diabetic conditions.

Genera	Trends	Wilcoxon rank sum one-sided tests, p-values
Lactobacillus	Decreasing in diabetes	0.06147082
Veillonella	Decreasing in diabetes	0.06986272
Tannerella	Increasing in diabetes	0.000134221
Bacteroidales_(G-2)	Increasing in diabetes	0.001046188
Parvimonas	Increasing in diabetes	0.002738778
Dialister	Increasing in diabetes	0.003268975
Pseudopropionibacterium	Increasing in diabetes	0.004440028
Fusobacterium	Increasing in diabetes	0.004621653
Saccharibacteria_(TM7)_(G-2)	Increasing in diabetes	0.004667303
Catonella	Increasing in diabetes	0.004681898
Treponema	Increasing in diabetes	0.008071337
Veillonellaceae_(G-1)	Increasing in diabetes	0.008112772
Corynebacterium	Increasing in diabetes	0.008718515
Saccharibacteria_(TM7)_(G-5)	Increasing in diabetes	0.009832632
Bacteroidetes_(G-6)	Increasing in diabetes	0.009835065
Gemella	Increasing in diabetes	0.010204779
Desulfovibrio	Increasing in diabetes	0.014608285
Peptoniphilaceae_(G-1)	Increasing in diabetes	0.015137114
Gracilibacteria_(GN02)_(G-1)	Increasing in diabetes	0.017135036
Lautropia	Increasing in diabetes	0.017658286
Acidipropionibacterium	Increasing in diabetes	0.022937793
Absconditabacteria_(SR1)_(G-1)	Increasing in diabetes	0.026786563
Campylobacter	Increasing in diabetes	0.028404684
Bergeyella	Increasing in diabetes	0.029533064
Cardiobacterium	Increasing in diabetes	0.036885103
Alloprevotella	Increasing in diabetes	0.043445848
Peptostreptococcaceae_(XI)(G-1)	Increasing in diabetes	0.044758275
Porphyromonas	Increasing in diabetes	0.046442212
Clostridiales_(F-1)(G-1)	Increasing in diabetes	0.047600272
Peptoniphilus	Increasing in diabetes	0.052798757
Saccharibacteria_(TM7)_(G-4)	Increasing in diabetes	0.052798757
Saccharibacteria_(TM7)_(G-8)	Increasing in diabetes	0.052798757
Peptostreptococcaceae_(XI)(G-5)	Increasing in diabetes	0.053284558
Saccharibacteria_(TM7)_(G-3)	Increasing in diabetes	0.054898561
Kingella	Increasing in diabetes	0.057678631
Lachnospiraceae_(G-3)	Increasing in diabetes	0.05810471
Aggregatibacter	Increasing in diabetes	0.059643116
Lachnospiraceae_(G-8)	Increasing in diabetes	0.060029912
Peptostreptococcaceae_(XI)(G-2)	Increasing in diabetes	0.064469595
Capnocytophaga	Increasing in diabetes	0.066144317
Saccharibacteria_(TM7)_(G-6)	Increasing in diabetes	0.072861614
Peptostreptococcus	Increasing in diabetes	0.073053921

Sneathia	Increasing in diabetes	0.076746012
Saccharibacteria_(TM7)_(G-1)	Increasing in diabetes	0.080774107
Anaerococcus	Increasing in diabetes	0.095127099
Cutibacterium	Increasing in diabetes	0.095127099
Enterobacter	Increasing in diabetes	0.095127099
Gracilibacteria_(GN02)_(G-2)	Increasing in diabetes	0.095127099
Mollicutes_(G-1)	Increasing in diabetes	0.095127099
Peptoniphilaceae_(G-2)	Increasing in diabetes	0.095127099
Propionibacteriaceae_(G-2)	Increasing in diabetes	0.095127099
Ruminococcaceae_(G-2)	Increasing in diabetes	0.095589402
Eggerthia	Increasing in diabetes	0.099245913
