# Supplementary material for: Alterations in the Oral Microbiome Associated With Diabetes, Overweight, and Dietary Components
Source: Front Nutr. 2022 Jul 6;9:914715. doi: 10.3389/fnut.2022.914715 (PMC9298547; doi:10.3389/fnut.2022.914715)
Supplement: Supplementary file 5 [file Table_4.pdf]

Table S4. Relationships between oral and systemic health status and oral microbial community structure. All counts or mean values were shown with percentages or standard deviations, respectively.

	SL1 (N=34)	SL2 (N=33)	SL3 (N=32)
**Smoking**			
no	15 (44.1%)	16 (48.5%)	18 (56.3%)
yes	19 (55.9%)	17 (51.5%)	14 (43.8%)
**Mouth condition (score)**	2.47 (±0.748)	2.52 (±0.619)	2.38 (±0.609)
**Denture wearing**			
no	1 (2.9%)	0 (0%)	4 (12.5%)
yes	33 (97.1%)	33 (100%)	28 (87.5%)
**Dental visits**	3.76 (±0.554)	3.88 (±0.415)	3.88 (±0.336)
**Periodontal disease (score)**	3.35 (±0.849)	3.24 (±0.751)	3.31 (±0.738)
**Difficulty of eating**			
no	6 (17.6%)	8 (24.2%)	13 (40.6%)
yes	28 (82.4%)	25 (75.8%)	19 (59.4%)
**Flow rates**	0.195 (±0.152)	0.245 (±0.222)	0.231 (±0.133)
**Oral health (score)**	1.24 (±0.855)	1.00 (±0.791)	0.969 (±0.967)
**BMI**	29.6 (±5.28)	30.0 (±5.49)	27.4 (±3.72)
**Diabetes**			
no	14 (41.2%)	19 (57.6%)	20 (62.5%)
yes	20 (58.8%)	14 (42.4%)	12 (37.5%)

SL: salivatypes are distinct communities of bacteria in saliva, identified by unsupervised clustering (see text). SL1 was specifically enriched in T2D subjects, whereas SL2 was specifically enriched in obese subjects. SL3 was highest among lean and non-T2D subjects.
