# Supplementary figures and images for: Alterations in the Oral Microbiome Associated With Diabetes, Overweight, and Dietary Components
Source: Front Nutr. 2022 Jul 6;9:914715. doi: 10.3389/fnut.2022.914715 (PMC9298547; doi:10.3389/fnut.2022.914715)

+ Fisher tests  $P < 0.1$

\* Fisher tests  $P < 0.05$

Log<sub>2</sub> OR

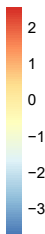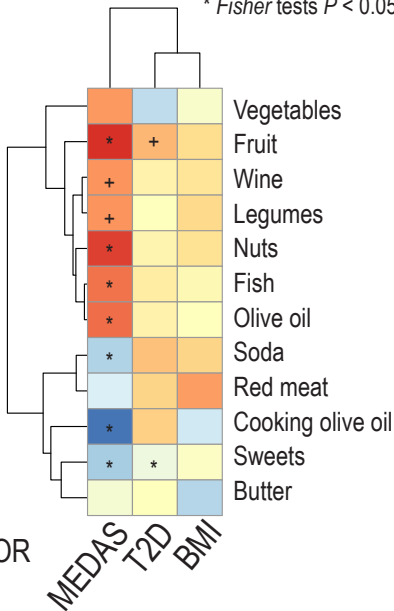

Supplement: FIGURE S1 — Odds ratios of intake food items by different MEDAS, T2D, and BMI status. (Fisher’s tests p-values shown) T2D: Type 2 diabetes mellitus. Note that a higher MEDAS score indicates a low consumption of sweet snacks (sweet), red meat, butter and soda and a high consumption of all other food items. [file Image_1.pdf]

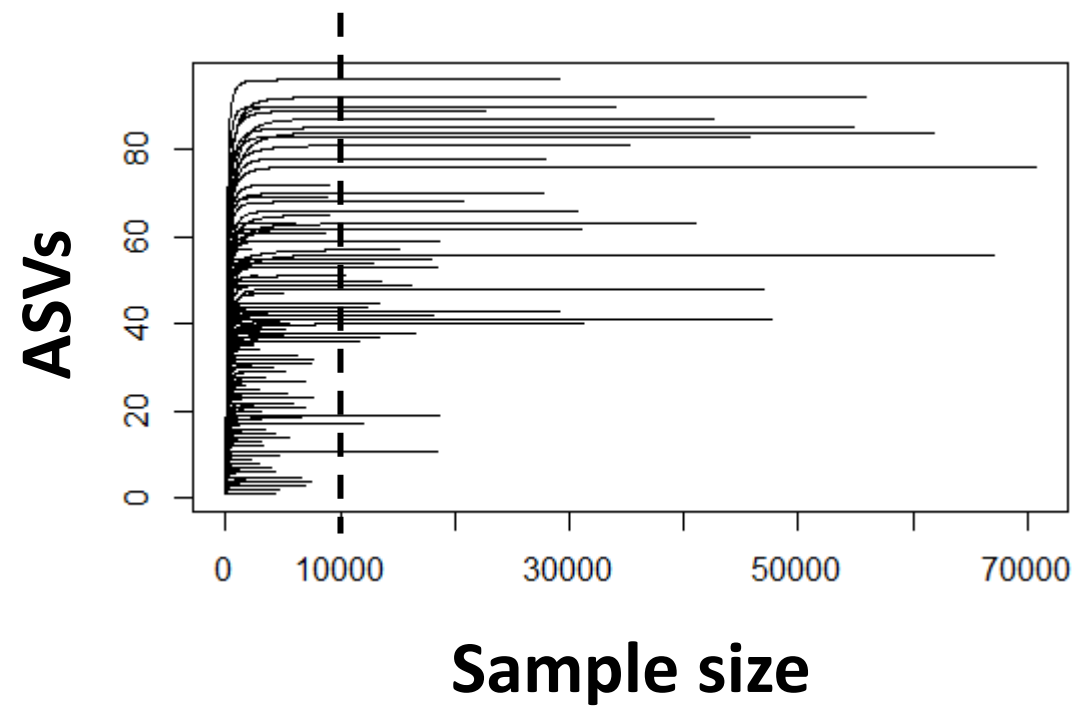

Supplement: FIGURE S2 — Rarefaction curves of amplicon sequence variants (ASVs), showing depth of 16SrRNA gene sequencing of saliva samples. [file Image_2.pdf]

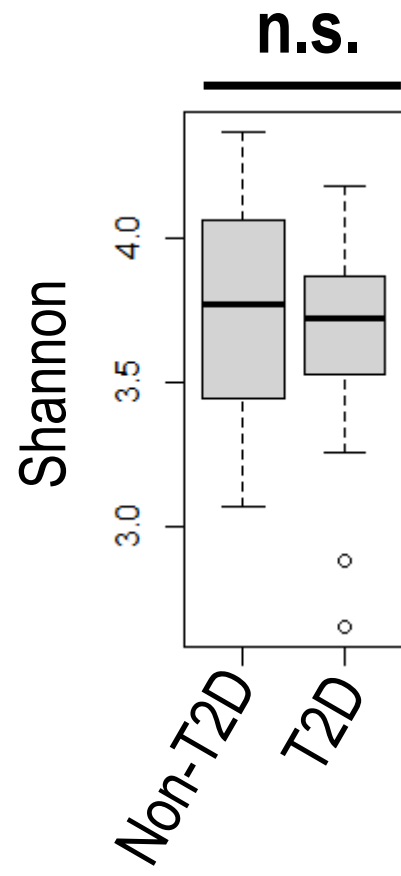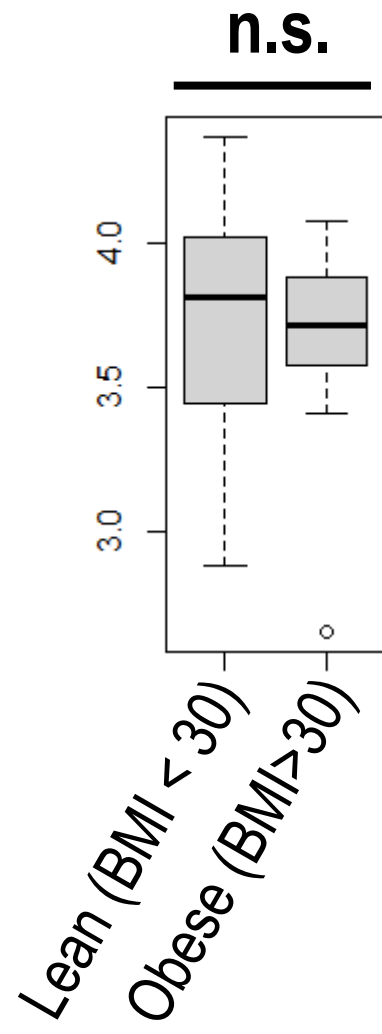

Supplement: FIGURE S3 — Salivatype-1 when present in non-T2DM, obese and non-obese subjects had a similar decreased alpha-diversity. [file Image_3.pdf]
